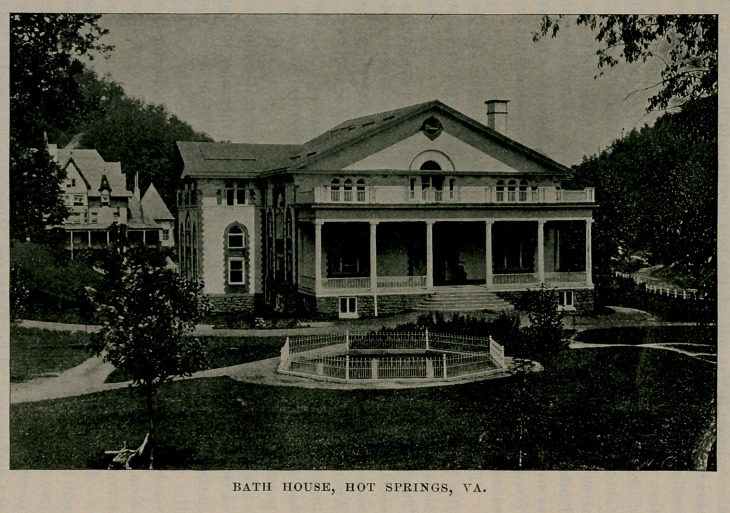# American Association of Obstetricians and Gynecologists

**Published:** 1896-11

**Authors:** 


### AMERICAN ASSOCIATION OF OBSTETRICIANS AND GYNECOLOGISTS.

THIS association held its ninth annual meeting at Richmond, September 22, 23 and 24, 1896, and it may be truthfully asserted that it was one of the best meetings in its history. This was the consensus of opinion among members upon adjournment, and as the published proceedings become available the judgment then expressed is reaffirmed.

In the first place, Richmond in late September is a most delightful city in which to hold such a meeting, and the hotel Jefferson, now available for such purposes, is second to no hostelry in the United States. We speak from a considerable personal experience as well as the judgment of others who have traveled extensively. The plan of the hotel is somewhat unique, its management is second to none, and its cuisine and furnishings are superb.

The local committee of arrangements, consisting of Dr. George Ben Johnston, chairman, Dr. Hugh M. Taylor, Dr. Edward McGuire, Dr. J. W. Long, Dr. J. F. Winn, Dr. Christopher Tompkins and Dr. Lewis C. Bosher deserve special credit for the excellent plans made and carried out for the entertainment of the association. It is difficult to see how they could have been better. At the instance of the committee the trunk lines of railways centering at Richmond conceded a reduction of passenger rates which bespeaks enterprise and liberality in the management of these important avenues of commerce and travel.

The Westmoreland, Commonwealth and Albemarle clubs opened their houses to the visitors, and most of the members and guests partook of their hospitalities. Drs. Taylor and McGuire entertained the association at a reception on one of the evenings, and Dr. George Ben Johnston appropriated another for that purpose, while Mrs. Christopher Tompkins received the ladies at her residence on one of the afternoons.

At the annual dinner of the association, which was served in one of the magnificent dining-rooms of the Jefferson on Wednesday evening, September 23d, a departure from the usual order was observed. In the first place, ladies were admitted to the dinner for the first time; and in the second, there were no speeches made in response to fixed toasts proposed. A reason for this change was because Mr. Polk Miller, of Richmond, who was the guest of the association, kindly offered to entertain it with his delineations of plantation negro life. The association thought the ladies who had accompanied members ought not to be deprived of the privilege of hearing Mr. Miller, and it was a pleasant change from the ordinary routine of toasts and speeches. Whoever has been fortunate enough to listen to Mr. Miller need not be told how

BATH HOUSE, HOT SPRINGS, VA.

entertaining he is, and whoever has not heard him has missed one of the most delightful and mirth-provoking entertainments that is given anywhere on the globe. No description can do it justice. It was an entertainment at once refined in character, charming in the method of its presentation and provocative of the highest sense of humor and pathos.

The Valentine Meat Juice Company entertained the association at luncheon at their abbatoir on the north side of Richmond, on the third day of the meeting. Here the members were afforded an opportunity to witness the scientific slaughter of beeves for the purpose of manufacturing meat juice, and to inspect one of the most complete and expensive abbatoirs in the country.

After the adjournment of the association, that is to say, Friday, September 25th, Dr. C. W. P. Brock, chief surgeon of the Chesapeake & Ohio railway, took the members of the association in charge for a tour as the guests of the railway, and under his direction a visit was first paid to the University of Virginia at Charlottesville. Here Professor Parkinson entertained the visitors at luncheon and a few hours were spent in strolling about the grounds and buildings of this historic old institution. In the evening a west-bound train was boarded and the visitors were taken to Hot Springs, where they enjoyed a rest until Sunday. This beautiful location in the mountains of Virginia is one of the most delightful resorts in the country. In addition to its princely hotel, The Homestead, it has a bath establishment that is unexceled, the cost of which was $150,000. After two days' rest the guests sped eastward and westward to their respective homes on the superb fast trains of the Chesapeake & Ohio railway, and so ended a most successful scientific meeting, which was also charmingly complete in its social entertainments.

## Figures and Tables

**Figure f1:**